# Human pancreatic islet microRNAs implicated in diabetes and related traits by large-scale genetic analysis

**DOI:** 10.1073/pnas.2206797120

**Published:** 2023-02-09

**Authors:** Henry J. Taylor, Yu-Han Hung, Narisu Narisu, Michael R. Erdos, Matthew Kanke, Tingfen Yan, Caleb M. Grenko, Amy J. Swift, Lori L. Bonnycastle, Praveen Sethupathy, Francis S. Collins, D. Leland Taylor

**Affiliations:** ^a^Center for Precision Health Research, National Human Genome Research Institute, NIH, Bethesda, MD 20892; ^b^British Heart Foundation Cardiovascular Epidemiology Unit, Department of Public Health and Primary Care, University of Cambridge, Cambridge CB2 0BB, UK; ^c^Heart and Lung Research Institute, University of Cambridge, Cambridge CB2 0BB, UK; ^d^Department of Biomedical Sciences, College of Veterinary Medicine, Cornell University, Ithaca, NY 14853

**Keywords:** pancreatic islets, diabetes, microRNA, eQTL

## Abstract

Genetic studies have identified ≥240 loci associated with the risk of type 2 diabetes (T2D), yet most of these loci lie in non-coding regions, masking the underlying molecular mechanisms. Recent studies investigating mRNA expression in human pancreatic islets have yielded important insights into the molecular drivers of normal islet function and T2D pathophysiology. However, similar studies investigating microRNA (miRNA) expression remain limited. Here, we present data from 63 individuals, the largest sequencing-based analysis of miRNA expression in human islets to date. We characterized the genetic regulation of miRNA expression by decomposing the expression of highly heritable miRNAs into *cis-* and *trans-*acting genetic components and mapping *cis*-acting loci associated with miRNA expression [miRNA-expression quantitative trait loci (eQTLs)]. We found i) 84 heritable miRNAs, primarily regulated by *trans*-acting genetic effects, and ii) 5 miRNA-eQTLs. We also used several different strategies to identify T2D-associated miRNAs. First, we colocalized miRNA-eQTLs with genetic loci associated with T2D and multiple glycemic traits, identifying one miRNA, miR-1908, that shares genetic signals for blood glucose and glycated hemoglobin (HbA1c). Next, we intersected miRNA seed regions and predicted target sites with credible set SNPs associated with T2D and glycemic traits and found 32 miRNAs that may have altered binding and function due to disrupted seed regions. Finally, we performed differential expression analysis and identified 14 miRNAs associated with T2D status—including miR-187-3p, miR-21-5p, miR-668, and miR-199b-5p—and 4 miRNAs associated with a polygenic score for HbA1c levels—miR-216a, miR-25, miR-30a-3p, and miR-30a-5p.

## Significance

Previous studies, mostly in animal models, suggest that microRNAs (miRNAs) play an important role in type 2 diabetes (T2D). Yet, we have limited knowledge of miRNA expression in human pancreatic islets (HPIs), a T2D-relevant tissue. Here, we present the largest sequencing-based analysis of miRNA expression in HPIs to date and evaluate islet miRNA expression in the context of T2D. We identify genetic effects that drive miRNA expression and show that highly heritable islet miRNAs are largely regulated by *trans*-effects. We also identify 14 miRNAs associated with T2D and 4 with a polygenic score for glycated hemoglobin. This study provides key insights into HPI miRNA biology and future pathways for investigating T2D pathophysiology.

Type 2 diabetes (T2D) is a leading contributor to global morbidity and mortality (1) and is characterized by reduced insulin response in insulin-sensitive tissues and pancreatic islet beta cell dysfunction (2, 3). However, despite our knowledge of the role of insulin in the disease, the underlying molecular and cellular mechanisms driving T2D are not wholly understood. Recent studies investigating circulating biomarkers of T2D identified several microRNAs (miRNAs) with altered levels in diabetic patients (4–9), raising the possibility that miRNAs play an important role in human T2D pathophysiology.

Mature miRNAs are small (~22 nucleotides long), non-coding RNA molecules that typically function as post-transcriptional gene regulators by tethering the miRNA-induced silencing complex to target messenger RNAs (mRNAs), leading to mRNA silencing and degradation (10–12). Several seminal studies have implicated miRNAs in pancreatic islet development and function (13–24). However, the bulk of these studies are in model systems (e.g., animal models, cell lines), so our knowledge of miRNAs in human pancreatic islets (HPIs) remains limited. To date, most studies examining miRNA expression in HPIs have tested only a small number of miRNAs (25–28). Of the two more comprehensive sequencing-based studies of HPI miRNAs (29, 30), both have been limited in sample size (n ≤ 7).

In addition, incorporating genetic information in the analysis of miRNA expression in HPIs may help identify miRNAs with a causal role in T2D pathophysiology. To date, ≥240 loci have been reported to be associated with T2D risk, yet the majority of these signals lie in non-coding regions of the genome, masking the underlying molecular mechanisms (31, 32). Previous studies have successfully integrated genetic and RNA-seq data in HPIs to characterize the genetic drivers of mRNA expression and nominate candidate causal genes for T2D by identifying shared genetic signals between loci associated with islet gene expression quantitative trait loci (eQTLs) and T2D risk (33–37). However, such integrative analyses have yet to be performed for miRNAs in HPIs.

**Fig. 1.**
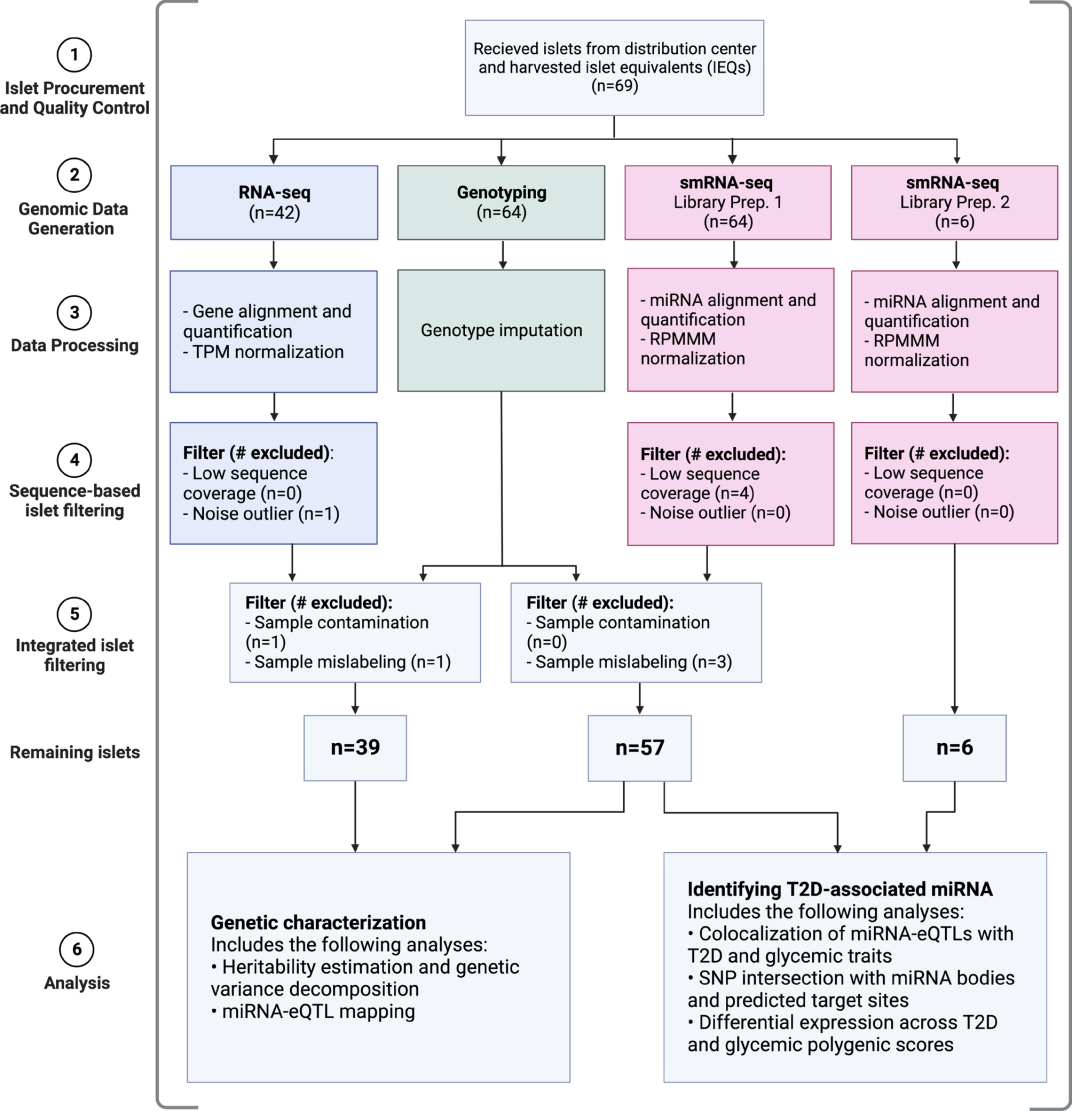
Study overview. Colors correspond to data modalities. TPM: transcripts per million; RPMMM: reads per million mapped to miRNAs. Created with BioRender.com.

Here, we present data from 63 individuals, the largest sequencing-based analysis of miRNA expression in HPIs to date (Fig. 1 and *SI Appendix*, Table S1; 57 with genotypes). Using genotype information (n = 57), we estimate the SNP-based heritability for each miRNA (i.e., the proportion of variation in expression that can be explained by additive effects of common genetic variants) and find 84 highly heritable miRNAs. To perform a comparative analysis of heritability between miRNAs and mRNAs, we also estimate the SNP-based heritability of mRNAs using a subset of islets with genotype and mRNA expression data from this study (n = 39) and a previous study (n = 189) (33). We show that regulation of heritable miRNAs is primarily driven by *trans*-acting genetic effects, whereas heritable mRNAs are regulated by a combination of *cis-*acting and *trans-*acting genetic effects. Given the importance of islet dysfunction in T2D pathophysiology, we apply several different strategies to assess the role of miRNA expression in diabetes. First, we map miRNA-eQTLs and colocalize the five identified miRNA-eQTLs with genetic signals from association studies of T2D and glycemic traits. Next, we identify single nucleotide polymorphisms (SNPs) from these genetic studies that are in the 99% credible sets (i.e., variants that are most likely to be the causal variant at each independent genetic association signal) and lie in miRNA seed regions or predicted target sites. Finally, using all miRNA expression data (n = 63), we identify miRNAs associated with T2D status and polygenic scores (PGSs) of T2D-related traits, as well as sex, age, and body mass index (BMI).

## Results

### Data Overview.

We procured 69 HPI samples and performed small RNA-sequencing (smRNA-seq), RNA-sequencing (RNA-seq), and genotyping, retaining 63 samples with smRNA-seq, 39 samples with RNA-seq, and 57 samples with genotypes after quality control steps (*Materials and Methods*; Fig. 1 and *SI Appendix*, Fig. S1 and Table S1). We note that not all samples were subject to each assay (Fig. 1). For the smRNA-seq, we generated data through two experiments: library preparation 1 (LP1; 57 samples) and library preparation 2 (LP2; 6 samples). In LP1, we generated an average of 38.87 million read pairs per sample (±14.45 SD; minimum read count ≥19.98 million), with a mean read length of 23.24 nucleotides (*SI Appendix*, Fig. S2*A*). In LP2, we generated an average of 64.36 million read pairs per sample (±4.18 SD; minimum read count ≥58.61 million), with a mean read length of 22.62 nucleotides (*SI Appendix*, Fig. S2*B*). Across both LPs, we found that the miRNAs identified by previous mouse and human studies (17, 29, 38) to be most abundant in islets (e.g., miR-375) were also the most abundant miRNAs in the data generated in this study (*SI Appendix*, Fig. S4). In total, we identified 2,959 unique miRNAs, including 1,989 miRNA isoforms (isomiRs). Of these, 2,279 are either canonical (reference) miRNAs or isomiRs with nucleotide shifts ≤2 in either direction, which we referred to as “high confidence.” For the RNA-seq, we generated an average of 57.51 million read pairs per sample (±16.48 SD; minimum read count ≥21.49 million). Similar to smRNA-seq, we found that highly abundant mRNAs (e.g., *PRSS1, REG1A*) from previous HPI studies (35, 37) were also highly abundant in the islet mRNA data generated in this study (*SI Appendix*, Fig. S5), underscoring the data quality.

### Heritability and Genetic Architecture of miRNA Expression.

**Fig. 2.**
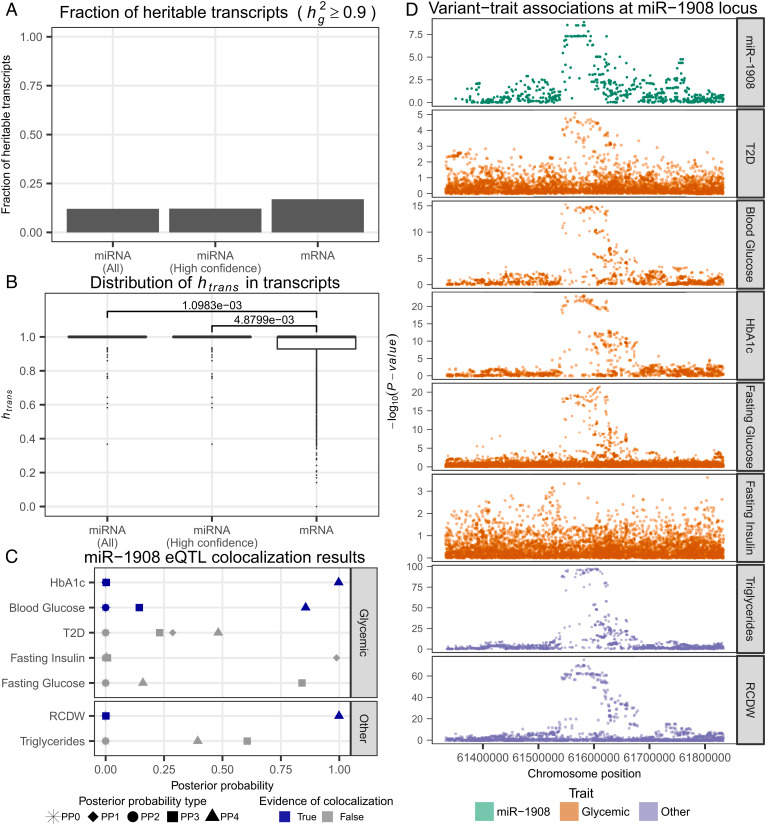
Genetic analysis of miRNA expression in HPIs. (*A*) Fraction of heritable transcripts ($h_{g}^{2}$ ≥ 0.9) across miRNAs and mRNAs. (*B*) Distribution of variance explained by *trans*-acting genetic effects in the expression of heritable transcripts. *P*-value calculated using Mann–Whitney *U* test. (*C*) Evidence of genetic colocalization between miR-1908 eQTL and disease/trait genetic association signals (*Materials and Methods*). Posterior probability definitions: PP0 – neither trait is associated, PP1 – miRNA-eQTL is associated, PP2 – Genome-wide association study (GWAS) is associated, PP3 – both traits are associated with different causal variants, PP4 – both traits are associated and share a single causal variant. (*D*) For each disease/trait used in the colocalization analysis, Manhattan plots for all variants within a 250 kb window on either side of the mature miR-1908 transcript.

Studies in mice hepatic and lung tissues (39, 40) suggest that the genetic architecture of miRNA expression is primarily driven by *trans*-acting factors, much more than mRNA which has a substantial *cis*-acting genetic component; however, to date, a comparison of miRNA and mRNA regulatory trends has yet to be explored in human islets. To compare the trends in genetic regulation of miRNA and mRNA species, we estimated the SNP-based heritability ($h_{g}^{2}$) for miRNA and mRNA transcripts using imputed genotypes of common SNPs (*Materials and Methods*). We found that the fraction of heritable miRNA transcripts ($h_{g}^{2}$ ≥ 0.9) was substantially smaller than the fraction of heritable mRNA transcripts for both all miRNA transcripts (*P* = 8.70 × 10^−4^, Chi-squared test; Fig. 2A and *SI Appendix*, Fig. S6*A*) and high-confidence miRNA transcripts (*P* = 1.56 × 10^−3^, Chi-squared test; Fig. 2A and *SI Appendix*, Fig. S6*A*), suggesting that miRNAs are under stronger selection pressure than mRNAs (*SI Appendix*, Note S1).

We sought to understand the genetic architecture of heritable transcripts and decomposed the variance in transcript expression into *cis-* or *trans-*acting genetic effects, assuming that proximal SNPs [i.e., SNPs found within 250 kb of the mature miRNA transcript or mRNA transcription start site (TSS)] function primarily via *cis*-acting mechanisms and distal SNPs (i.e., outside of a 250 kb window) function primarily via *trans*-acting mechanisms, as has been shown previously (41). We found that the variance explained by *trans*-effects (*h_trans_*) was greater in heritable miRNA transcripts than in heritable mRNA transcripts (*P* = 1.10 × 10^−3^, Mann–Whitney *U* test; Fig. 2B and *SI Appendix*, Fig. S6*B*). These trends held true when just considering high-confidence miRNA transcripts (*P* = 4.88 × 10^−3^, Mann–Whitney *U* test; Fig. 2B and *SI Appendix*, Fig. S6*B*) as well as a larger 20 Mb *cis* window size, to account for the fact that some miRNA TSSs may be quite far away from the miRNA body (*P* = 1.00 × 10^−2^, Mann–Whitney *U* test; *SI Appendix*, Fig. S8) (42). To ensure these results were not driven by the limited sample size of the mRNA dataset (n = 39), we repeated the analysis using an independent dataset of 189 HPI samples with mRNA expression and genetic data from a previous study (33) and replicated these findings (*SI Appendix*, Fig. S9 *A* and *B*; *P* = 2.75 × 10^−14^, Chi-squared test for comparative heritability analysis; *P* = 3.75 × 10^−2^, Mann–Whitney *U* test for *trans*/*cis* variance decomposition). Combined, these results suggest the genetic regulatory architecture of miRNAs differs from mRNAs as miRNA genetic regulation is primarily driven by *trans*-effects while mRNA is driven by a combination of *trans-* and *cis-*effects. Such a model is consistent with previous studies in rodents (39, 40).

### Identification of Genetic Associations with miRNA Expression.

We tested for genetic associations with miRNA expression in order to identify miRNA-eQTLs (*Materials and Methods*). At a false discovery rate (FDR) of ≤5%, we found five miRNA-eQTLs (*SI Appendix*, Table S3). We performed colocalization analysis between these miRNA-eQTLs and genetic loci associated with T2D and glycemic traits, including fasting blood glucose levels, blood glucose levels, fasting insulin levels, and glycated hemoglobin (HbA1c; *Materials and Methods*). We found no colocalizations with T2D. However, for one miRNA-eQTL, an eQTL for miR-1908 tagged by rs174559, we found evidence of colocalization with HbA1c and blood glucose levels (Fig. 2 *C* and *D*). Since rs174559 is also strongly associated with triglycerides (TG) (43) and red blood cell distribution width (RCDW, a metric representing the heterogeneity of red blood cell volume) (44), we also performed colocalization with these traits. We found no evidence of colocalization with TG, but evidence of colocalization with RCDW (Fig. 2*C*), possibly suggesting pleiotropic effects of this locus across tissues.

Focusing on the islet-relevant colocalization with glycemic traits, we looked for i) *cis-*effects of rs174559 on the transcription of nearby protein coding genes in HPIs and ii) *trans*-effects of rs174559 on protein coding genes throughout the genome to identify candidate target transcripts of miR-1908. We performed colocalization analysis between the miRNA-eQTL signal for miR-1908 and genetic associations for gene and exon expression identified in an islet eQTL study spanning 420 individuals (33). We found evidence of colocalization with several *FADS1* exons and with gene level *FADS1* expression (*SI Appendix*, Fig. S11*A*). Although miR-1908 is located within a *FADS1* exon (there are several *FADS1* isoforms), we found that the strongest colocalization signal was for variants associated with an exon ~4.3 kb away from miR-1908. We performed Mendelian randomization and meditation analyses to test for a potential causal relationship between miR-1908 and *FADS1* (or vice versa; *Materials and Methods*). We found no evidence for a causal relationship between miR-1908 and *FADS1* expression (*SI Appendix*, Fig. S11*B*). Next, we looked for *trans*-associations between rs174559 and protein coding genes in 228 samples [39 samples from this study and 189 samples from an independent dataset (33)] as *trans-*eQTL summary statistics from previous islet eQTL studies were not available (*Materials and Methods*). We found no associations, which may be driven by no signal or the lack of power to detect *trans*-eQTL effects at this sample size.

Previous studies have mapped miRNA-eQTLs in blood (45, 46). To understand the tissue specificity of miRNA genetic effects, we compared the effect sizes of SNP-miRNA pairs from HPI miRNA-eQTLs to blood miRNA-eQTLs (*Materials and Methods*). We found that although some effects are replicated across datasets [e.g., the HPI rs174559 miR-1908 eQTL is also found in the study by Sonehara et al. (46)], the HPI effects were generally not correlated with the genetic effects observed in blood (*SI Appendix*, Fig. S12; maximum Spearman’s rho = 0.07), suggesting that genetic effects on miRNAs may to some extent be tissue/cell type specific.

### Identification of T2D-Related Genetic Effects that May Perturb miRNA Function.

To identify genetic variants associated with T2D and T2D-related phenotypes that may affect islet miRNA function (as opposed to overall expression levels), we overlapped 99% credible set SNPs from genetic studies for T2D and other glycemic traits with i) mature miRNA genomic coordinates (which include areas outside of the seed region and may affect the ability of the miRNA to bind to targets) and ii) genomic coordinates for predicted miRNA target regions (i.e., the miRNA binding site on the mRNA transcript). To limit the analysis to islet-relevant results, we retained SNPs where the miRNA and target mRNA were highly expressed in HPI data presented in this study (*Materials and Methods*; *SI Appendix*, Table S4). Consistent with previous work (47), we did not identify any SNPs within mature miRNA coordinates. However, we did identify 16 T2D, 8 HbA1c, and 1 blood glucose credible set SNPs within predicted miRNA target regions. Because SNPs within miRNA target regions would exert their effects on the target transcript via *cis*-acting mechanisms, we further explored the effects of these SNPs using an islet *cis*-eQTL study spanning 420 individuals (33). We found one SNP, rs1464569—in the 99% credible set for an HbA1c signal—that lies within *NICN1* at a miR-532-3p target site and is in high linkage disequilibrium (1000GENOMES phase_3 FIN = 1.00, CEU = 1.00, GBR = 1.00) with the tag SNP, rs4955440, for the gene-level *NICN1* eQTL. To explore whether rs1464569 functions as an eQTL for *NICN1* by perturbing miRNA binding, we tested for an interaction effect by modeling *NICN1* expression with rs1464569 and miR-532-3p expression using 33 samples with mRNA expression, miRNA expression, and genetic data (*Materials and Methods*). We found no evidence of an interaction effect (*P* > 0.05), leaving the mechanism driving the genetic association at this locus unresolved.

### Differentially Expressed miRNAs.

Next, we sought to identify miRNAs associated with sex, age, BMI, T2D disease status, and PGSs for T2D and glycemic traits (*Materials and Methods*).

**Fig. 3.**
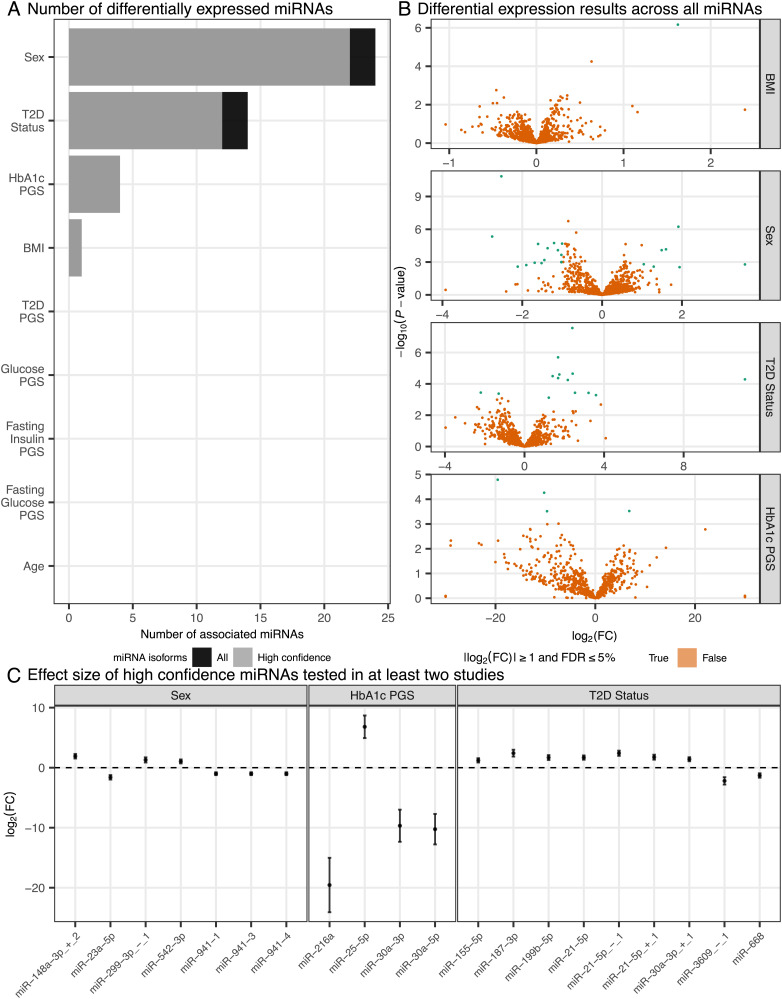
Differentially expressed miRNAs across diseases/traits. (*A*) Number of differentially expressed miRNAs (FDR ≤ 5% and |log_2_(FC)| ≥ 1) across each disease/trait. (*B*) Volcano plots describing the log_2_(FC) (*x* axis) and -log_10_(*P*-value) (*y* axis) of miRNAs across BMI, sex (male vs. female), T2D status, and HbA1c PGS. (*C*) Forest plot describing the effect of high-confidence differentially expressed miRNAs (FDR ≤ 5% and |log_2_(FC)| ≥ 1). Effect sizes are from meta-analysis.

For sex, age, and BMI, we performed a meta-analysis of differential expression results from this study (n = 63 total from two LPs) and a previous smRNA-seq study of HPIs (n = 7) (29). We identified 1 BMI-associated and 24 sex-associated miRNAs [FDR ≤ 5% and |log_2_(fold change (FC)]| ≥ 1; Fig. 3 and *SI Appendix*, Figs. S13 and S14). The large number of sex-associated miRNAs is consistent with a previous study describing substantial genomic differences between male and female HPIs (28). Notably, all but two (miR-542-3p and miR-767-5p) of the sex-associated miRNAs occurred on autosomes.

Similarly, for T2D status, we compared T2D (n = 4) to normal glucose-tolerant (NGT, n = 59) donors and meta-analyzed the differential expression results from this study with a previous study (29) consisting of 7 islet samples (n = 4 T2D, n = 3 NGT). We identified 14 miRNAs with altered expression in T2D (FDR ≤ 5% and |log_2_(FC)| ≥ 1), 13 of which were not identified previously in the study by Kameswaran et al. (Fig. 3 and *SI Appendix*, Fig. S15), including 12 high-confidence miRNAs such as miR-21-5p, miR-187-3p, miR-199b-5p, miR-668, and miR-4497.

For the PGSs, we used all miRNA samples with genotypes (n = 57) and meta-analyzed differential expression results from individuals of European (n = 42), African (n = 7), and Hispanic/Latino (n = 6) ancestries. We identified four miRNAs associated with the HbA1c PGS (FDR ≤ 5% and |log_2_(FC)| ≥ 1; Fig. 3 and *SI Appendix*, Fig. S16)—miR-216a, miR-25-5p, miR-30a-5p, and miR-30a-3p.

Across all of the phenotypes considered, we compared the overlap of associated miRNAs (*SI Appendix*, Fig. S17). We found that one miRNA, miR-122-5p, was associated with both T2D and BMI.

To establish mRNAs that may be regulated by the miRNAs identified by our differential expression and genetic analyses, we tested for associations between miRNA expression and the mRNA expression of i) all genes and ii) only the predicted target genes (from TargetScan) using 33 samples with mRNA and miRNA expression data (*Materials and Methods*). At an FDR ≤ 5%, we identified no associations, likely driven by limited power with only 33 samples.

Finally, previous studies describe cell type heterogeneity as a possible confounder for bulk tissue gene expression studies (48, 49). To assess the contribution of cell type heterogeneity to the results described in this study, we estimated the fraction of alpha and beta cells, the primary cell types in processed HPIs (50–52), using miRNA profiles of sorted alpha and beta cells (*Materials and Methods*; *SI Appendix*, Fig. S20) (29). For both the miRNA-eQTL (*SI Appendix*, Fig. S21*A*) and differential expression analyses (*SI Appendix*, Fig. S21 *B*–*E*), we observed little change in the results of the models, suggesting cell type heterogeneity is not a primary driver of the results reported in this study.

## Discussion

MiRNAs are small, non-coding RNA molecules that serve as important post-transcriptional gene regulators in human health and disease [reviewed in the study by Singh and Storey (53)]. The critical contribution of miRNAs to proper pancreatic islet development and function as well as the dysregulation of miRNAs in the context of T2D is well documented by previous studies using rodent models and cell-based systems (13–24). Despite these advances, our knowledge of miRNA expression and activity in human islets remains limited.

In this study, we present the largest sequencing-based analysis of miRNA expression in HPIs to date (n = 63). We describe the genetic regulation of HPI miRNA expression and provide insight into the role of miRNAs in T2D pathophysiology.

In our genetic analysis of HPIs, we show that the expression of highly heritable miRNAs is driven primarily by *trans*-acting genetic effects, whereas the expression of highly heritable mRNAs is driven by a combination of *cis-* and *trans-*acting genetic effects. This model is supported by previous studies in rodents that compare the heritability of miRNAs and mRNAs (39, 40) and suggests that miRNAs may be under greater selective pressure than mRNAs, as has been postulated by previous studies in humans (47, 54). Such selection pressures imply that to map miRNA-eQTLs thoroughly, large sample sizes will be required. Although this study replicates a previous finding, it is important to note that the heritability estimates of miRNAs and mRNAs will be affected by differences in signal-to-noise dynamics, making a direct comparison between the two features challenging (55) (*SI Appendix*, *Note* S1). We attempt to minimize the effects of noise by using noisyR (56) to remove low-abundance, noisy transcripts (*Materials and Methods*). However, in order to confidently compare the heritability between two biological phenomena measured by two different assays, future studies using repeat sampling of replicates will be required (55, 57).

Among the five identified miRNA-eQTLs, we find one miRNA-eQTL (miR-1908 tagged by rs174559) that colocalizes with genetic signals for multiple physiological traits—blood glucose, HbA1c, and RCDW. This signal also colocalizes with a genetic association for gene- and exon-level expression of *FADS1*, an essential enzyme for long-chain polyunsaturated fatty acid synthesis that has been implicated in a range of diseases, including diabetes and related glycemic traits (58–62). However, we highlight three limitations. First, given the sample size of this study and the number of expected recombination events, our power to detect colocalization events is limited, so the lack of colocalizations for five miRNA-eQTLs does not rule out possible colocalizations at larger sample sizes (63). Second, it is unclear whether the identified miR-1908 effects are mediated by miR-1908, *FADS1*, both, or neither. We perform an analysis to disentangle directionality between the two transcripts, but we find no evidence of a causal relationship between miR-1908 and *FADS1*, suggesting that the genetic effect may be independent. Further experiments to understand the molecular effects at this locus will be required. Finally, it is unclear whether the HbA1c and blood glucose effect is mediated by islets, blood, or some other tissue type. On the one hand, given i) the rs174559 miR-1908 eQTL also occurs in blood (46), ii) the RCDW effect at this locus, and iii) extensive studies that document how erythrocytic (i.e., red blood cell related) genetic effects can mediate HbA1c levels (64–68), it is plausible that observed effect on HbA1c is mediated by blood. On the other hand, an erythrocytic mediated effect would not explain the observed blood glucose genetic effect. In addition, at this locus, a study partitioned HbA1c genetic associations into erythrocytic and glycemic effects (69). Using a SNP in high linkage disequilibrium with rs174559 (1000GENOMES phase_3 FIN = 0.67, CEU = 0.70, GBR = 0.71) (70, 71), this study determined that the effect at rs174559 on HbA1c was driven by glycemic effects rather than erythrocytic effects which would suggest that the observed effects in islets may indeed be relevant.

As part of our genetic analysis, we also intersect 99% credible set SNPs with the genomic coordinates of predicted miRNA target sites. We find one SNP, rs1464569, in the 99% credible set for an HbA1c signal that lies in the *NICN1* target site for miR-532-3p and is in high linkage disequilibrium with the tag SNP for the islet *NICN1* eQTL. This finding may indicate that the eQTL functions by perturbing miR-532-3p regulation of *NICN1.* However, when we tested for an interaction effect in *NICN1* expression, we did not find evidence of an interaction effect as would be expected if the *NICN1* eQTL functioned by perturbing miR-532-3p binding. *NICN1* is a tumor suppressor gene that has been shown to promote cell differentiation in a cell line derived from nasopharyngeal carcinomas (72); to date, little is known about the function of this gene in HPIs—a topic for future studies.

In addition, we identify several HPI miRNAs differentially expressed across BMI, sex, T2D status, and HbA1c genetic scores. Of the miRNAs identified, 26 (61.9%) have been previously implicated at some level as being associated with a related phenotype (38, 73–89). Focusing on the miRNAs associated with T2D status and HbA1c genetic scores, these findings provide critical human islet-based confirmation of previous studies in rodents and cell lines. For example, we find HPI levels of miR-216a and miR-25 to be negatively and positively correlated with HbA1c genetic scores, respectively. In previous work, the miR-216a knockout mouse was reported to have defects in insulin secretion in the context of chronic high-fat diet (90), and miR-25 has been described as a negative regulator of insulin synthesis in INS-1 cells, a rat insulinoma cell line (75). As another example, we show that miR-199b-5p is associated with T2D status, consistent with a previous study in murine beta cells where miR-199b-5p was shown to be highly responsive to glucose stimulation (91). We also find that miR-21-5p is strongly associated with T2D status. This observation comports with a recent study in which overexpression of miR-21-5p led to reduced glucose-stimulated insulin secretion in mice and human beta cells (92). We report that increased HPI expression of one miRNA, miR-122-5p, is associated with both increased BMI and T2D status. These findings are consistent with a previous study that reports that increased expression of miR-122-5p in blood is associated with T2D status and increased circulating insulin levels (81). As a final example, we identify the expression of miR-668, previously reported to be associated with T2D in human skeletal muscle (87), as a newly identified T2D association in HPIs.

Across all of the miRNAs identified by our genetic analysis and differential expression analysis, we tested for associations with potential target mRNAs, but found no associations (FDR ≤ 5%), likely driven by the small sample size of samples with paired miRNA and mRNA data (n = 33). Future studies with multiomic readouts or targeted miRNA perturbations paired with mRNA sequencing will be required to understand the target genes of the identified miRNAs.

Unraveling the genetic factors that contribute to pancreatic islet function and T2D is a high priority for the diabetes research community. However, much of the focus has been on mRNA and chromatin. Here, we add data on another important source of molecular regulation. While this is the largest sequencing-based analysis of HPI miRNA expression to date, larger studies will be required to probe the genetics of HPI miRNAs more comprehensively and to uncover more subtle associations between miRNA expression and phenotypes of interest. Of particular importance is increasing the sample size of islets from T2D patients, as the current sample size of T2D patients analyzed (n = 8) is still small. Nonetheless, the findings and associations identified in this study are a step toward understanding HPIs in the context of diabetes and will help guide the prioritization of miRNAs for future mechanistic studies.

## Materials and Methods

A detailed description of computational and experimental analyses is provided in **SI Appendix*, Materials and Methods*. Briefly, we performed smRNA-seq on 63 HPI samples and RNA-seq on 39 HPIs—quantifying miRNA and mRNA expression. Using 63 HPIs with genotypes, we estimated the SNP-based heritability for miRNA (n = 57) and mRNA (n = 39) transcripts and decomposed the variance in the expression of heritable transcripts into *cis-* and *trans*-acting genetic components using LIMIX v3.0.4. We tested for genetic variants associated with miRNA expression (miRNA-eQTLs) using LIMIX v3.0.4. To identify T2D-relevant miRNAs, we performed a colocalization analysis with miRNA-eQTLs and genetic loci associated with T2D and glycemic traits using coloc v3.1. To identify potential gene targets of miRNAs that colocalized with T2D or a glycemic trait, we also performed a colocalization analysis with miRNA-eQTLs and genetic loci associated with exon and gene expression in HPIs. We overlapped 99% credible set SNPs from genetic studies for T2D and glycemic traits with miRNA mature transcripts and target sites to identify variants that may alter miRNA function. Finally, we used DESeq2 v1.32.0 to identify miRNAs differentially expressed across T2D status, PGSs of T2D and glycemic traits, and other common phenotypes (i.e., sex, age, and BMI).

## Supplementary Material

Appendix 01 (PDF)

## Data Availability

Anonymized data deposits: the individual-level genotype, RNA-seq, and LP1 smRNA-seq data generated in this study are available in the database of Genotypes and Phenotypes (dbGap) with accession no. phs001188.v2.p1 (93); data are accessible through dbGaP’s standard data access request procedures. The individual-level LP2 smRNA-seq data from this study are available in the Gene Expression Omnibus data repository with accession no. GSE196797 (94). Summary statistics for the differential expression and miRNA-eQTL analyses are available in the Zenodo data repository with accession no. 7516377 (95).
